# The nasopharyngeal microbiome in South African children with lower respiratory tract infection: a nested case-control study of the Drakenstein Child Health Study

**DOI:** 10.21203/rs.3.rs-4605876/v1

**Published:** 2024-07-19

**Authors:** Shantelle Claassen-Weitz, Yao Xia, Lesley Workman, Luke Hannan, Sugnet Gardner-Lubbe, Kilaza S Mwaikono, Stephanie Harris Mounaud, William C. Nierman, Samantha Africa, Fadheela Patel, Felix Sizwe Dube, Veronica Allen, Lemese Ah Tow Edries, Heather J. Zar, Mark Patrick Nicol

**Affiliations:** University of Cape Town; University of Western Australia; Red Cross War Memorial Children’s Hospital; University of Cape Town; Stellenbosch University; Dar es Salaam Institute of Technology; J. Craig Venter Institute; J. Craig Venter Institute; University of Cape Town; University of Cape Town; University of Cape Town; University of Cape Town; University of Cape Town; University of Cape Town; University of Western Australia

**Keywords:** 16S rRNA gene, bacterial, infant, low- and middle-income country, lower respiratory tract infection, microbiome, nasopharyngeal, qPCR, viral

## Abstract

**Background:**

Lower respiratory tract infection (LRTI) is a leading cause of infant morbidity and mortality globally. LRTI may be caused by viral or bacterial infections, individually or in combination. We investigated associations between LRTI and infant nasopharyngeal (NP) viruses and bacteria in a South African birth cohort.

**Methods:**

In a case-control study of infants enrolled in the Drakenstein Child Health Study (DCHS), LRTI cases were identified prospectively and age-matched with controls from the cohort. NP swabs were tested using quantitative real-time polymerase chain reaction (qPCR) and 16S rRNA gene amplicon sequencing. We calculated adjusted Conditional Odds Ratios (aORs) for qPCR targets and used mixed effects models to identify differentially abundant taxa between LRTI cases and controls and explore viral-bacterial interactions.

**Results:**

Respiratory Syncytial Virus (RSV) [aOR: 5.69, 95% CI: 3.03–10.69], human rhinovirus (HRV) [1.47, 1.03–2.09], parainfluenza virus [3.46, 1.64–7.26], adenovirus [1.99, 1.08–3.68], enterovirus [2.32, 1.20–4.46], *Haemophilus influenzae* [1.72, 1.25–2.37], *Klebsiella pneumoniae* [2.66, 1.59–4.46], or high-density (> 6.9 log_10_ copies/mL) *Streptococcus pneumoniae* [1.53, 1.01–2.32] were associated with LRTI. Using 16S sequencing, LRTI was associated with increased relative abundance of Haemophilus (q = 0.0003) and decreased relative abundance of *Dolosigranulum* (q = 0.001), *Corynebacterium* (q = 0.091) and *Neisseria* (q = 0.004). In samples positive for RSV, *Staphylococcus* and *Alloprevotella* were present at lower relative abundance in cases than controls. In samples positive for parainfluenza virus or HRV, *Haemophilus* was present at higher relative abundance in cases.

**Conclusions:**

The associations between bacterial taxa and LRTI are strikingly similar to those identified in high-income countries, suggesting a conserved phenotype. RSV was the major virus associated with LRTI. *H. influenzae* appears to be the major bacterial driver of LRTI, acting synergistically with viruses. The Gram-positive bacteria *Dolosigranulum* and *Corynebacteria* may protect against LRTI, while *Staphylococcus* was associated with reduced risk of RSV-related LRTI.

**Funding:**

National Institutes of Health of the USA, Bill and Melinda Gates Foundation, National Research Foundation South Africa, South African Medical Research Council, L’Oréal-UNESCO For Women in Science South Africa, Australian National Health and Medical Research Council.

## BACKGROUND

Lower respiratory tract infection (LRTI) remains the leading cause of child death outside of the neonatal period worldwide [1]. The burden of LRTI disproportionately affects low- and middle-income countries (LMICs), with more than 200 deaths per 100,000 children under the age of five years in 2019 in several countries in Sub-Saharan Africa [2]. Viruses, particularly Respiratory Syncytial Virus (RSV), and bacteria not included in vaccines, particularly non-type b *Haemophilus influenzae*, are considered to play an increasingly important role [1].

Infection of the upper respiratory tract with pathogenic viruses and bacteria frequently precedes LRTI; with local replication and subsequent translocation to the lower respiratory tract [3]. Dysbiotic (imbalanced) NP bacterial community profiles may have reduced capability to resist pathogen overgrowth and invasion. For example, LRTI caused by RSV has been associated with increased abundance of H. *influenzae* and *Streptococcus* species and decreased abundance of *Staphylococcus* aureus [4]. *H. influenzae* and *Streptococcus*-dominated profiles have also been associated with an exaggerated inflammatory response and more severe RSV disease [4].

Risk factors for LRTI, such as malnutrition, maternal HIV-infection, lack of exclusive breastfeeding and indoor air pollution, are more prevalent in LMICs compared to high-income countries, which may influence aetiology and pathogen interactions. However, studies investigating the association between infant NP bacterial community profiles, viral infection and LRTI have primarily been performed in high-income settings. We therefore performed a case-control study, nested within a South African birth cohort, to comprehensively investigate associations between NP bacterial community profiles, viral infection and LRTI within the first year of life.

## METHODS

### Study design, participants, and specimen matching:

We conducted a case-control study of infants enrolled in the Drakenstein Child Health Study (DCHS), a birth cohort study in a peri-urban area in South Africa [5]. Ethical approval was received from the Human Research Ethics Committee (HREC) of the University of Cape Town, South Africa (401/2009 and 585/2015). Mothers were enrolled in their 2nd trimester of pregnancy; all births and hospital care occurred at a central public hospital [5]. Mother-infant pairs were followed from birth, with study visits at 6, 10 and 14 weeks, and 6, 9 and 12 months. Participants who gave additional consent participated in intensive fortnightly NP sampling, across the first year of life. Demographic and clinical data were recorded antenatally at birth, and postnatally during scheduled study visits (Appendix p. 2).

LRTI episodes during the first year of life were identified by active surveillance at local clinics and hospital using World Health Organisation (WHO) criteria [6]. Mothers were counselled about key respiratory symptoms and advised to contact study staff whenever their infant developed cough or difficulty breathing [6]. Infants were followed up through hospitalization or ambulatory illness. LRTI cases and non-LRTI controls were matched 1:1 by birth date and study site.

NP flocked swabs (Copan Diagnostics, CA, USA) were collected fortnightly from infants across the first year of life (0–365 + 14 days) (Figure S1). A window of +/−14 days was allowed between LRTI episode date and specimen collection date for inclusion as a LRTI case specimen. Additional information regarding the categorization of case-control specimens is provided in Appendix (pp. 3–4; Figure S1).

### Laboratory procedures

NP specimens were immediately suspended in PrimeStore^®^ Molecular Transport medium (Longhorn Vaccines & Diagnostics, MD, USA), transported on ice and subsequently stored at −80°C. Nucleic acid extracts (Appendix p. 4) were screened for viral and bacterial species using a quantitative real-time polymerase chain reaction (qPCR) [Fast-Track Diagnostics Respiratory Pathogens 33 assay, Luxembourg] [7]. We applied a predefined threshold (> 6.9 log_10_ copies/mL) for defining high density *Streptococcus pneumoniae* that best differentiates case-control status, as previously described [8].

We performed short read 16S rRNA gene amplicon sequencing on NP specimens from a subset of case-control specimens from whom sufficient sample was available. Each sequencing run included a comprehensive set of sequencing controls alongside NP specimens (Appendix pp. 4–5; Figure S2) [9]. We measured total 16S rRNA gene copy numbers (16S rRNA gene copies/μl) from nucleic acid extracts via qPCR [9]. A two-step PCR protocol targeting the V4 hypervariable region of the 16S rRNA gene was used to generate 16S rRNA gene amplicons. Pooled libraries were sequenced on the Illumina^®^ MiSeq^™^ platform using V3 chemistry with 2 × 251 cycles [MiSeq Reagent Kit v3 (600-cycle) Reagent Cartridge (Illumina, CA, USA)] (Appendix pp. 5–7).

We used the DADA2 pipeline (wrapped in the Nextflow algorithm) to filter and trim reads and infer amplicon sequence variants (ASVs) (Appendix p. 7). Taxonomy was assigned using the RDP classifier implementation for DADA2 and SILVA v138. We used R software v4.1.2 [10] and RStudio v2021.09.2 to remove ASVs classified as Eukaryota and ASVs unclassified at Kingdom-level. We applied a step-wise in-silico quality control approach to remove low quality amplicon data [9] and potential contaminant ASVs (Appendix p. 8).

### Outcomes

The primary outcome was the association between viral or bacterial species (qPCR), or bacterial taxa (16S rRNA gene amplicon sequencing), in cases with LRTI compared to age-matched controls.

### Statistical analysis

We calculated Conditional Odds Ratios (ORs) and Population Attributable Fractions (PAFs) using Miettinen’s formula for each viral and bacterial organism included in the qPCR panel. We further stratified results within three age categories: 0–3 months, > 3–6 months, and > 6–12 months. Findings were adjusted for age and antibiotic use.

Associations between LRTI and NP bacterial load or alpha diversity (Shannon and Chao1) were assessed using linear regression. PERMANOVA (Bray-Curtis dissimilarity assessed using adonis2 function from the vegan package) was used to determine if overall microbial composition differed between cases and controls [11]. Regression and PERMANOVA variables included case status, age and antibiotic exposure.

K means clustering was used to identify specimen clusters, based on the profiles of the 25 most abundant ASVs. We used logistic regression to explore associations between cluster membership and case status. The model included cluster membership, absolute abundances of RSV (A/B), parainfluenza virus, enterovirus, adenovirus, HRV, cytomegalovirus (CMV), age and bacterial load.

We used the Microbiome Multivariable Associations with Linear Model (MaAsLin2) v1.8.0 [12] to identify differentially abundant ASVs between cases and controls. Mixed effects models, with participant identifier as a random effect and case-control group, age, and antibiotic exposure as fixed effects were used. Total sum scaled (TSS) normalization and log transformation were applied. The results were visualized using Seaborn v0.12 [13] in Python. Subsequently, using identical settings, we chose four subsets comprising individuals infected with RSV, HRV, parainfluenza virus or CMV, and applied the MaAsLin2 model to identify bacterial taxa associated with LRTI.

### Role of the funding source

The funders of the study had no role in study design, data collection, data analysis, data interpretation, or writing of the report.

## RESULTS

### Demographics and clinical characteristics

**Figure 1:**
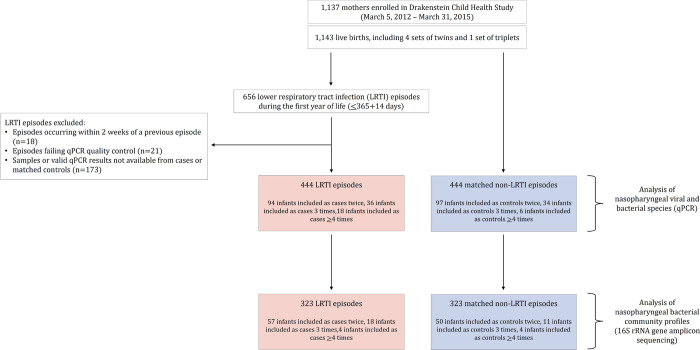
Study flow diagram

From 29 May 2012 to 3 September 2015, 1,137 mothers were enrolled with 1,143 live births. Cohort retention was high [88.8% (1,015/1,143) infants at 1 year] [14]. A total of 656 LRTI episodes were identified during the first year of life (Fig. 1). Results from qPCR were available for 444 case specimens and 444 matched control specimens (Fig. 1). A subset of these specimens [323 LRTI specimens and 323 control specimens] had high-quality 16S rRNA gene amplicon sequence data available (Appendix pp. 13–17).

Maternal smoking was identified in 27% (151/544) participants. Poor socio-economic status was reflected by low maternal educational attainment (67% of mothers had primary level education only), any parent employment rate (49.6%), and monthly income [< 5,000 ZAR per month for 88% of households]. The median household size was 4 people [interquartile range (IQR): 3–6]. Most infants were delivered by vaginal delivery (80%, 442/542) with 17% (93/544) born prematurely (< 37 weeks’ gestation); most [58/93 (62%)] late preterm. Twenty seven percent (148/544) of infants were HIV-exposed but none were HIV-infected, and duration of exclusive breastfeeding was short [1.4 months (IQR: 0.5–3.0)]. Immunization coverage for all expanded programme on immunization vaccines was high (> 98%) including for *H. influenzae* type B conjugate vaccine and 13-valent pneumococcal conjugate vaccine.

The median age of NP specimen collection from age-matched cases and controls at the time of LRTI was 139 days (IQR: 81–220 days) (Table 1). Nineteen percent (85/444) of case specimens were collected from cases who received antibiotics prior to specimen collection; none of the controls received antibiotics (p < 0.001).

### qPCR of nasopharyngeal pathogens associated with LRTI

**Figure 2:**
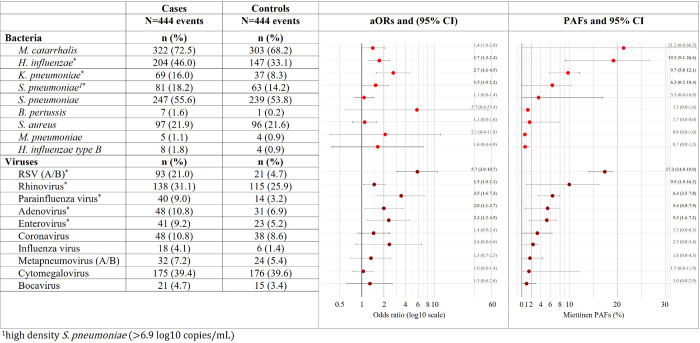
Associations between bacteria and viruses detected from nasopharyngeal (NP) specimens and lower respiratory tract infection (LRTI) during the first year of life Adjusted odds ratios (aORs) and population attributable fractions (PAFs) calculated for each of the NP pathogens screened using the Fast-Track Diagnostics Respiratory Pathogens 33 (FTDResp33) assay for 544 participants (444 LRTI and 444 non-LRTI specimens). Significant associations are denoted by an asterisk.

qPCR (n = 888 specimens) identified bacteria and viruses which were associated with LRTI (Fig. 2). Bacterial species associated with LRTI included *H. influenzae* [adjusted odds ratio (aOR): 1.72, 95% confidence interval (95% CI): 1.25–2.37], *Klebsiella pneumoniae* [aOR: 2.66, 95% CI: 1.59–4.46], and high-density *Streptococcus pneumoniae* [aOR: 1.53, 95% CI: 1.01–2.32] (Fig. 2).

Adjusted odds ratios (aORs) and population attributable fractions (PAFs) calculated for each of the NP pathogens screened using the Fast-Track Diagnostics Respiratory Pathogens 33 (FTDResp33) assay for 544 participants (444 LRTI and 444 non-LRTI specimens). Significant associations are denoted by an asterisk.

Among infants aged 0–3 months, bacteria significantly associated with LRTI were *H. influenzae*, *K. pneumoniae* and *S. pneumoniae* (Figure S4 A). Among children aged > 3–6 months, *H. influenzae* and *Moraxella catarrhalis* were associated with LRTI (Figure S5 A), while among children aged > 6–12 months, *K. pneumoniae* was associated with LRTI (Figure S6 A).

Viruses associated with LRTI during the first year of life included RSV [aOR: 5.69, 95% CI: 3.03–10.69], HRV [aOR: 1.47, 95% CI: 1.03–2.09], parainfluenza virus [aOR: 3.46, 95% CI: 1.64–7.26], adenovirus [aOR: 1.99, 95% CI: 1.08–3.68], and enterovirus [aOR: 2.32, 95% CI: 1.20–4.46] (Fig. 2). RSV, CMV and parainfluenza virus were significantly associated with LRTI among children aged 0–3 months (Figure S4 A), while HRV and RSV were associated with LRTI among children > 3–6 months (Figure S5 A) and RSV was associated with LRTI among children > 6–12 months (Figure S6 A-B). Results of qPCR in the subset of participants from whom 16S rRNA gene amplicon sequence data were available (n = 646 specimens) corresponded with the full dataset (Figure S3).

The population attributable fraction (PAF) reflects the proportion of LRTI which may be attributed (alone or in combination) to a particular pathogen. For RSV, which was both prevalent and strongly associated with LRTI, the PAF was 17.3%, 95% CI: 14.0–19.0 (Fig. 2). Other organisms which contributed substantially to LRTI aetiology were *H. influenzae* [PAF 19.2%, 95% CI: 9.1–26.6], *K. pneumoniae* [PAF 9.7%, 95% CI: 5.8–12.1], and HRV [PAF 9.9%, 95% CI: 1.0–16.2].

### Investigation of bacterial community profiles associated with LRTI

Following quality control steps (Fig. 1; Appendix p. 13–17), a total of 323 age- and site-matched case-control sets (n = 646 specimens) were included. The median read count per specimen was 19,283 (IQR: 13,022–25,220). Following the removal of potential contaminant amplicon sequence variants (ASVs) (Table S1), a total of 826 ASVs were included, 98% of which were classifiable at genus-level.

**Figure 3:**
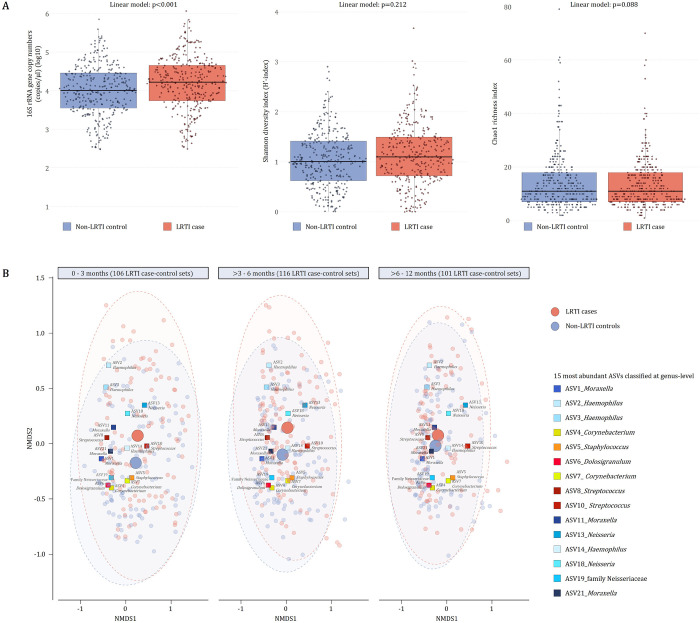
Bacterial density and diversity measured from NP specimens collected from LRTI cases and controls A) Bacterial density (16S rRNA gene copies/μl) (left panel) and within-specimen bacterial diversity [Shannon diversity (middle panel) and Chao1 richness (right panel)] compared between nasopharyngeal (NP) specimens collected from lower respiratory tract infection (LRTI) cases and controls. P-values are derived from linear models adjusted for specimen collection age and antibiotic therapy prior to specimen collection. Median values are presented by horizontal lines within each of the boxplots while upper and lower ranges of the boxplots represent the 75% and 25% quartiles, respectively. Maximum and minimum values, excluding outliers, are shown by whiskers. B) Non-metric Multi-Dimensional Scaling (NMDS) plots of Bray-Curtis dissimilarity showing loadings for the 15 most abundant amplicon sequence variants (ASVs) present in the dataset. ASVs are denoted using multicoloured squares. Shades of multicoloured squares are used to present phylum-level classification of each of the ASVs [shades of blue: Proteobacteria, shades of yellow: Actinobacteria, shades of red: Firmicutes]. The three NMDS plots (left, middle and right) show NP specimens collected at 0–3 months, >3–6 months, and >6–12 months, respectively. Small red and blue circles represent NP specimens collected from LRTI cases and controls, respectively. Large red and blue circles represent centroids of LRTI cases and controls, respectively. The number of LRTI case-control sets included in each age interval are shown at the top of each NMDS plot. Alpha bags enclosing 90% of samples are shown for LRTI cases and controls.

NP bacterial load (16S rRNA gene copies/μl) was higher in specimens from cases compared with controls (p < 0.0001) (Fig. 3A). Lower NP bacterial load was observed in specimens from infants who had antibiotic therapy prior to specimen collection, compared with infants with no antibiotic therapy (≤ 24 hours: p = 0.004, > 24 hours ≤ 7 days: p < 0.001). NP microbiome alpha diversity (Shannon: p = 0.213, Chao1: p = 0.088) was not significantly different when comparing cases with controls (Fig. 3A). Higher alpha diversity was observed in specimens from infants who had received antibiotic therapy prior to specimen collection compared with those who had not (Shannon: p = 0.036 and Chao1: p = 0.011 for antibiotic therapy > 24 hours ≤ 7 days prior to specimen collection vs no antibiotics; Chao1: p = 0.026 for antibiotic therapy < 24 hours of collection vs no antibiotics). There were significant differences in overall NP microbiome composition between cases and controls (PERMANOVA: p < 0.001, PERMDISP: p = < 0.001) (Fig. 3B).

### Composition of the nasopharyngeal microbiome varies with age, antibiotic therapy and case-status

**Figure 4:**
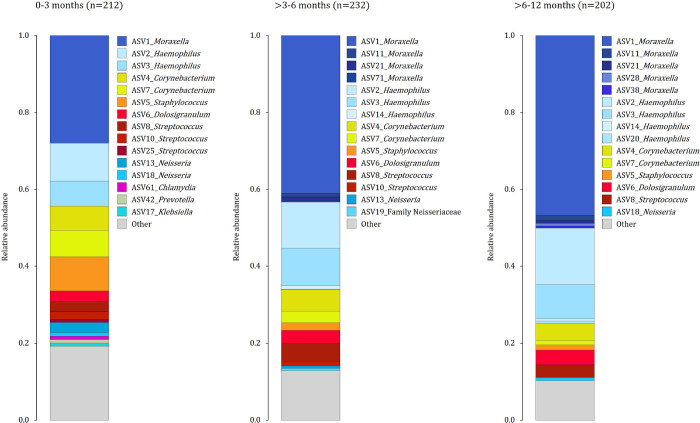
Barplots of compositional mean relative abundances of the top 15 amplicon sequence variants (ASVs) detected from nasopharyngeal (NP) specimens collected at 0–3 months (n=212), >3–6 months (n=232) and >6–12 months (n=202) The 15 most abundant ASVs from each age group are shown using colours representing phylum-level classification (Shades of blue: Proteobacteria; shades of yellow: Actinobacteria; shades of red: Firmicutes, shades of green: Bacteroidetes).

Compositional mean relative abundances of the top 15 ASVs in each age category are summarised in Fig. 4 and Table S2. Differential abundance testing [12] showed that participant age (Table S3), was strongly associated with relative abundance of several ASVs (including negative association between age and *Staphylococcus*, *Corynebacterium*, *Streptococcus*-ASV10 and positive association between age and *Moraxella, Haemophilus*, *Dolosigranulum* and *Streptococcus*-ASV8). Antibiotic therapy prior to specimen collection was associated with reduced relative abundance of *Dolosigranulum* and *Moraxella* and increased relative abundance of anaerobes, including *Alloprevotella*, *Porphyromonas*, and *Gemella*, as well as *Streptococcus* ASV10 and Family *Neisseriaceae* ASV19 (Table S4).

**Figure 5:**
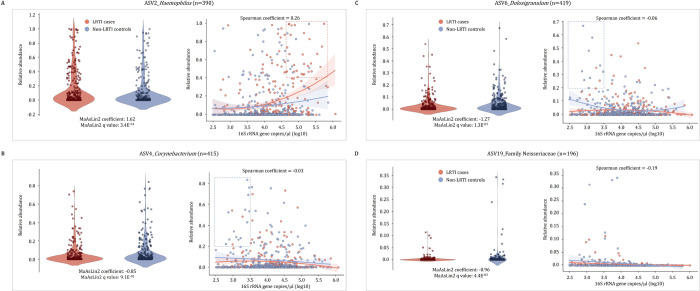
Relative abundances of amplicon sequence variants (ASVs) associated with LRTI case or control status Relative abundances of A) *Haemophilus* (ASV2), B) *Corynebacterium*(ASV4), C) *Dolosigranulum* (ASV6), and D) an unclassified ASV from the family Neisseriaceae (ASV19) detected from nasopharyngeal (NP) specimens collected from lower respiratory tract infection (LRTI) cases and controls. Violin plots on the left of each plot show the distribution of relative abundances for each of the ASVs detected from NP specimens collected from LRTI cases and controls. Differential abundance testing results (q values and coefficients) using Microbiome Multivariable Associations with Linear Models (MaAsLin2) are shown for LRTI case-control status. ASVs with p values <0.05 and q values <0.25 were considered significantly differentially abundant. MaAsLin2 linear models were adjusted for age at specimen collection and antibiotic therapy prior to specimen collection. Scatter plots of relative abundances of each ASV plotted against bacterial load (16S rRNA gene copies/μl) are shown on the right of each plot, with specimens collected from LRTI cases shown in red and controls in blue. Trendlines represent the trends estimated by LOESS (locally estimated scatterplot smoothing). Shaded areas represent 95% confidence intervals.

When adjusting for age and antibiotic therapy, specimens from cases had significantly higher relative abundances of *Haemophilus* (ASV2) when compared to controls (Fig. 5A). NP specimens with both high bacterial load (> 50,000 16S rRNA gene copies/μl) and high relative abundances (> 40%) of *Haemophilus* (ASV2) were primarily collected from LRTI cases (77%, 27/35) (Fig. 5A) (McNemar test: p < 0.0001). In contrast, *Corynebacterium* (ASV4), *Dolosigranulum* (ASV6), and an unclassified genus of the Family Neisseriaceae (ASV19) were detected at significantly higher relative abundances in specimens from controls compared to cases (Fig. 5B–D). NP specimens with both low bacterial load (< 3,000 16S rRNA gene copies/μl) and high relative abundances (> 20%) of *Corynebacterium* (ASV4) or *Dolosigranulum* (ASV6) were primarily collected from controls (81%, 13/16, McNemar test: p < 0.0001 or 81%, 9/11, McNemar test: p < 0.0001 respectively).

Relative abundances of A) *Haemophilus* (ASV2), B) *Corynebacterium* (ASV4), C) *Dolosigranulum* (ASV6), and D) an unclassified ASV from the family Neisseriaceae (ASV19) detected from nasopharyngeal (NP) specimens collected from lower respiratory tract infection (LRTI) cases and controls. Violin plots on the left of each plot show the distribution of relative abundances for each of the ASVs detected from NP specimens collected from LRTI cases and controls. Differential abundance testing results (q values and coefficients) using Microbiome Multivariable Associations with Linear Models (MaAsLin2) are shown for LRTI case-control status. ASVs with p values < 0.05 and q values < 0.25 were considered significantly differentially abundant. MaAsLin2 linear models were adjusted for age at specimen collection and antibiotic therapy prior to specimen collection. Scatter plots of relative abundances of each ASV plotted against bacterial load (16S rRNA gene copies/μl) are shown on the right of each plot, with specimens collected from LRTI cases shown in red and controls in blue. Trendlines represent the trends estimated by LOESS (locally estimated scatterplot smoothing). Shaded areas represent 95% confidence intervals.

### Analysis of possible viral-bacterial interactions using qPCR and short read 16S rRNA gene amplicon sequencing

**Figure 6:**
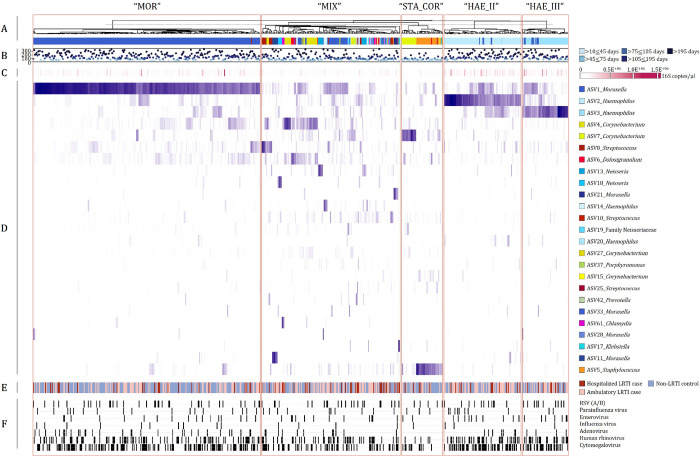
K-means clustering of relative abundances of the top 25 amplicon sequence variants (ASVs) detected from LRTI case-control nasopharyngeal (NP) specimens (n=646) A) Dendogram representing unsupervised hierarchical clustering distances of nasopharyngeal (NP) specimens based on relative abundances of the top 25 amplicon sequence variants (ASVs) in the dataset (n=646). NP specimens were grouped into five clusters: “MOR”, “STA_COR”, “HAE_II”, “HAE_III” and “MIX”. The most abundant ASVs from each specimen are shown using colours representing phylum-level classification (Shades of yellow: Actinobacteria; shades of green: Bacteroidetes; shades of red: Firmicutes, shades of blue: Proteobacteria). B) NP specimen collection age (in days). C) 16S rRNA gene amplicon copy numbers (copies/μl) measured from each NP specimen. D) Heatmap of relative abundances of the top 25 ASVs present in the dataset. E) Participant status (hospitalized LRTI case, ambulatory LRTI case or control). F) Presence/absence data of viruses screened using multiplex qPCR.

K-means clustering was used to group NP specimens (n = 646) into five clusters (Fig. 6). “MOR” cluster (n = 278) included NP specimens dominated by ASV1 (*Moraxella*). “STA_COR” cluster (n = 50) included NP specimens dominated by ASV5 (Staphylococcus) or ASV7 (*Corynebacterium*). “HAE_II” cluster (n = 94) included NP specimens dominated by ASV2 (*Haemophilus*), with variable contribution from ASV1 or ASV3 (*Haemophilus*). “HAE_III” cluster (n = 56) included specimens dominated by ASV3 with variable contribution from ASV1. “MIX” cluster (n = 168) was dominated by a range of other ASVs.

The distribution of age, case status and viral detection by cluster membership are shown in Table S6. We modelled the association between cluster membership and case status, controlling for age, bacterial load, and viral detection (Table S7). Compared with membership of cluster HAE_II, membership of cluster STA_COR was negatively associated with case status (estimate − 0.72, p = 0.001).

**Figure 7:**
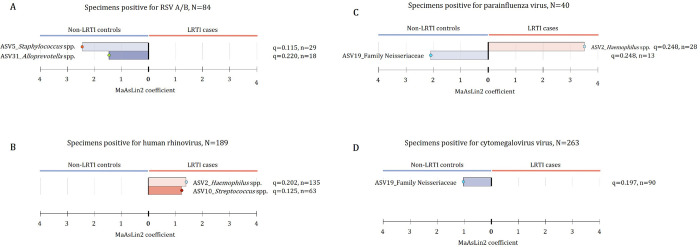
Differential abundance testing for viral and bacterial pathogens detected using multiplex qPCR Bacterial taxa which were associated with case-status (LRTI case vs. control, using linear regression models adjusted for age and antibiotic therapy prior to specimen collection) are shown for subsets of NP specimens positive on multiplex qPCR for A) respiratory syncytial virus (RSV), B) human rhinovirus (HRV), C) parainfluenza, and D) cytomegalovirus (CMV).

To explore whether the microbiome is associated with development of LRTI in infants infected with a respiratory virus, we investigated which bacterial taxa were associated with case status among infants infected with a particular virus. Among the subset of infants in whom RSV was detected (n = 84, 68 cases and 16 controls), we observed significantly lower relative abundances of *Staphylococcus* spp. (ASV5) and *Alloprevotella* spp. (ASV31) in cases when compared to controls (Fig. 7A). Among infants in whom HRV was detected (n = 189, 103 cases and 86 controls), we observed lower abundances of *Moraxella* spp. (ASV1) in cases than controls, and higher abundances of *Haemophilus* spp. (ASV_2) and *Streptococcus* spp. (ASV10) in cases (Fig. 7B). Similarly, in infants with parainfluenza virus (n = 40, 30 cases and 10 controls), *Haemophilus* spp. (ASV_2) relative abundance was higher among cases compared to controls.

Bacterial taxa which were associated with case-status (LRTI case vs. control, using linear regression models adjusted for age and antibiotic therapy prior to specimen collection) are shown for subsets of NP specimens positive on multiplex qPCR for A) respiratory syncytial virus (RSV), B) human rhinovirus (HRV), C) parainfluenza, and D) cytomegalovirus (CMV).

## DISCUSSION

We investigated associations between NP bacterial community profiles, viral infection and LRTI in South African infants enrolled in a birth cohort through the first year of life. Using qPCR, we found that viruses RSV, HRV, parainfluenza virus, adenovirus, and enterovirus, and the bacteria *Haemophilus influenzae*, *K. pneumoniae* and *S. pneumoniae* (high density colonization) were associated with LRTI. Using 16S rRNA gene amplicon quantification and sequencing, LRTI was associated with increased bacterial load, higher relative abundances of *Haemophilus,* and lower relative abundances of the commensal taxa Corynebacterium, *Dolosigranulum* and an unclassified genus within the family Neisseriaceae. Interactions between viruses and bacteria positively associated with LRTI included HRV with *Haemophilus* or *Streptococcus* and parainfluenza virus with *Haemophilus*, while the interaction between RSV and *Staphylococcus* or *Alloprevotella* was negatively associated with LRTI.

Our findings of a positive association of LRTI with *Haemophilus* and a negative association with *Corynebacterium* and *Dolosigranulum* via 16S rRNA gene amplicon sequencing are strikingly similar to those observed in children with upper or lower respiratory infection in the United States [15], Australia [16], Europe [17] and Botswana [18]. This finding therefore represents a conserved phenotype associated with respiratory infection across both high and middle-income settings and identifies key bacterial targets for prophylactic or therapeutic intervention. We did not find evidence that NP bacterial diversity was associated with LRTI. This is in contrast to previous findings that acute upper respiratory infections such as acute otitis media [19] and mucosal inflammation in chronic rhinosinusitis [20] are associated with a decrease in local bacterial diversity.

We have previously reported on the association between the detection of microbes, using qPCR, in the NP of children in this cohort and LRTI [21, 22]. Here, we report on a larger, partially overlapping cohort, which enabled age-stratification of findings. In keeping with our previous findings,[6, 7] we detected significant associations between LRTI and detection of viruses (RSV, HRV, parainfluenza virus and adenovirus) and bacteria (*H. influenzae, S. pneumoniae and K. pneumoniae*). Our findings are also similar to those previously reported in the multicenter PERCH study, but 16S microbiome analysis was not done [23]. A new finding from this study is that CMV was strongly associated with LRTI in the first three months of life, while *M. catarrhalis* was associated with LRTI between three and six months.

RSV contributed to a substantial proportion of LRTI cases (PAF = 17%) and was strongly enriched among cases. In contrast, the contribution of the highly prevalent *H. influenzae, M. catarrhalis* and HRV to LRTI is less straightforward to interpret. These three organisms were only weakly (although significantly) associated with LRTI, and were commonly detected in healthy children, suggesting that interactions between these organisms and other pathogens or host factors may be required for progression to LRTI. We therefore explored possible interactions between viruses and bacteria.

Among children in whom RSV was detected, the relative abundance of *Staphylococcus* or *Alloprevotella* was lower in children with LRTI compared with controls. Several reports have identified lower prevalence or abundance of *Staphylococcus* in children with RSV infection, compared with controls [4, 24]. Neutrophilic inflammation in the respiratory tract has been shown to predict symptomatic RSV disease [25], and S. *aureus* nasal colonization impairs neutrophil recruitment [26], suggesting that *S. aureus* colonization may reduce the risk of symptomatic RSV infection.

Among those infants infected with HRV or parainfluenza virus, *Haemophilus* relative abundance was higher among children with LRTI compared with controls. This is in keeping with the hypothesis that for viruses with relatively low pathogenicity, co-infection with bacteria, particularly *H. influenzae*, is important in driving progression to LRTI. Previous reports have identified that *H. influenzae* or S. pneumoniae infection may drive more severe illness in children with RSV LRTI, [4, 27], however our cohort included primarily children with mild LRTI, and we did not identify associations between the major *Haemophilus* ASVs (ASV2 or ASV3) or Streptococcus and RSV disease.

The role of *Moraxella* species remains unclear. *Moraxella*-ASV1 was the most abundant ASV detected, in keeping with our previous finding that *Moraxella* dominates the NP microbiota of infants [28]. qPCR targeting the species *M. catarrhalis* showed a positive association with LRTI, however, the genus *Moraxella* was not associated with LRTI overall, and, in children with HRV infection, was more abundant in controls than in cases. ASV1 also includes the understudied species *M. nonliquefasciens*, and it is possible that the balance between these two species of *Moraxella* in the NP influences associations with LRTI.

Although we excluded congenital pneumonia cases from our analysis, we identified an association between nasopharyngeal detection of CMV and LRTI in the first three months of life. CMV viraemia has been described in infants with hypoxic pneumonia [29], although whether CMV contributes to respiratory illness or whether illness triggers CMV viraemia is unclear. Autopsy studies have also identified CMV as an important contributory cause to death in children under the age of five years [30].

The combination of high bacterial load and high relative abundance of *Haemophilus* was strongly predictive of LRTI, while low bacterial load and high relative abundance of *Corynebacterium* or *Dolosigranulum* was associated with health. This supports the hypothesis that translocation of large numbers of opportunistic pathogens from the nasopharynx to the lower respiratory tract may be key to pathogenesis of LRTI, and that a low density microbiome dominated by these Gram-positive species may be protective.

Our study has several key strengths, including a robust birth cohort study design which reduces the risk of bias in identifying cases and controls, high cohort retention, strong surveillance for LRTI, large sample size and detection of viruses and bacteria using highly multiplexed qPCR as well as 16S rRNA gene amplicon sequencing. There are some limitations. First, although this is among the largest studies of its kind, our ability to study virus-bacteria interactions was limited for less prevalent viruses. Second, due to the cross-sectional study design, causal inferences are not possible. Third, while the NP niche is a reasonable proxy for the lower respiratory tract [31], we were unable to directly sample the lung due to the need for invasive sampling methods. A limitation of short-read 16S rRNA gene amplicon sequencing is taxonomic resolution. Finally, host responses including immune and metabolic profiles are required to understand host-microbe interactions in LRTI.

## CONCLUSIONS

The associations between bacterial taxa and LRTI are strikingly similar to those identified in high-income countries, suggesting a conserved phenotype. RSV was the major virus associated with LRTI. *H. influenzae* appears to be the major bacterial driver of LRTI, acting synergistically with viruses. The Gram-positive bacteria *Dolosigranulum* and Corynebacteria may protect against LRTI, while *Staphylococcus* was associated with reduced risk of RSV-related LRTI. Since patterns of microbial detection associated with LRTI are conserved across different continents and across varying income levels, interventions to reduce the global burden of LRTI should address these conserved patterns. With the implementation of new passive immunisation prevention against RSV in the first six months of life, active immunisation strategies and new interventions targeting non-typeable *H. influenzae* should be prioritized to reduce the burden of LRTI. Treatments, such as probiotics, should be explored to enhance natural protection associated with Gram-positive commensals. Mechanisms through which Gram-positive bacterial colonization of the nasopharynx may reduce the risk of viral LRTI require further study.

## Figures and Tables

**Table 1:** Characteristics of DCHS study population included in the LRTI case control analysis.

	qPCR data	16S rRNA gene amplicon data^ϕ^
	N = 544	N = 479
	n (%)	n (%)
Maternal smoking (self-report):		
Yes	151 (27.3)	127 (26.5)
Maternal education:		
Primary level	52 (9.4)	43 (9.0)
Started secondary level	319 (57.6)	277 (57.8)
Completed secondary level	151 (27.3)	132 (27.6)
Any tertiary level	32 (5.8)	27 (5.6)
Parent employed:		
Yes	275 (49.6)	237 (49.5)
Household income (per month):		
< 1,000 ZAR^i^	187 (33.8)	160 (33.4)
1,000–5,000 ZAR	302 (54.5)	264 (55.1)
> 5,000 ZAR	65 (11.7)	55 (11.5)
Household density, median (IQR^ii^)	4 (3–6)	4 (3–6)
Mode of delivery:		
Vaginal delivery	442 (80.1)^2^	382 (79.9)^1^
Season of birth:		
Summer	152 (27.4)	129 (26.9)
Autumn	152 (27.4)	136 (28.4)
Winter	134 (24.2)	114 (23.8)
Spring	116 (20.9)	100 (20.9)
WAZ^iii^ at birth, median (IQR)	−0.3 (−0.0–0.4)	−0.2 (−0.9–0.4)
Gestational age:		
Premature (< 37 weeks’ gestation)	93 (16.8)	72 (15.0)
Sex:		
Male	294 (53.1)	252 (52.6)
HIV^iv^-exposure:		
HIV-exposed, uninfected	148 (26.7)	127 (26.5)
Exclusive breastfeeding (months), median (IQR)	1.4 (0.5–3.0)^10^	1.1 (0.5–2.8)
Vaccine coverage		
Birth (BCG, OPV)	545 (99.1)^4^	472 (99.2)^3^
6 weeks (OPV, RV, DTaP-IPV-Hib-HepB, PCV13)	548 (99.6)^4^	474 (99.6)^3^
10 weeks (DTaP-IPV-Hib-HepB)	548 (99.6)^4^	474 (99.6)^3^
14 weeks (RV, DTaP-IPV-Hib-HepB, PCV13)	545 (99.5)^6^	471 (99.4)^5^
9 months (DTaP-IPV-Hib-HepB, PCV13)	523 (97.8)^19^	452 (97.8)^17^
Specimen-level data		
	qPCR data	16S rRNA gene amplicon data^ϕ^
	N = 888	N = 646
	n (%)	n (%)
Age at specimen collection (days), median (IQR)	139 (81–220)	128 (75–211)
Antibiotic use at specimen collection		
No	799 (90.0)	581 (89.9)
Antibiotic commenced < 24 hours prior to specimen collection	55 (6.2)	41 (6.4)
Antibiotic commenced > 24 hours and < 7 days prior to specimen collection	30 (3.4)	24 (3.7)
Unknown	4 (0.4)	0 (0.0)

Superscript values represent missing values (n)

ϕ Subset of qPCR data

i ZAR: South African Rand

ii IQR: interquartile range

iii WAZ: Weight-for-age z-score

iv HIV: Human immunodeficiency virus

v BCG: Bacillus Calmette-Guerin

vi OPV: Oral polio vaccine

vii RV: Rotavirus vaccine

viii DTaP-IPV-Hib-HepB: Diphtheria-tetanus-acellular pertussis-injectable polio-*Haemophilus influenzae* b-Hepatitis B vaccine

ix PCV13: 13-valent Pneumococcal conjugate vaccine

## Data Availability

qPCR data is available at https://github.com/xy-repo/Drakenstein-Nested-Case-Control. The 16S rRNA gene sequencing data supporting the conclusions of this article will be made available in the National Centre for Biotechnology Information (NCBI) Sequence Read Archive (SRA). Metadata included in this study is available at https://github.com/xy-repo/Drakenstein-Nested-Case-Control/raw_data/metadata_323sets_final.csv. This paper reports original code for MaAsLin2 analyses, which is available in https://github.com/xy-repo/Drakenstein-Nested-Case-Control.

## Research in context

### Evidence before this study

We searched PubMed on 8 February 2024, using the search terms “child” AND (“pneumonia” OR “lower respiratory tract infection”) and “microbiome” and “virus”. We identified case-control or cohort studies from high-income countries and a study from Botswana studying the bacterial component of the microbiome with or without associated viral detection. Most studies identified that the bacterial genera *Haemophilus* and *Streptococcus* were associated with lower respiratory tract infection (LRTI), while *Corynebacterium* and *Dolosigranulum* were associated with health. Viruses, including RSV, influenza, parainfluenza, HRV and human metapneumovirus were frequently associated with infection.

### Added value of this study

To our knowledge, this is the largest study incorporating both multiplex viral detection and 16S rRNA gene sequencing in infants with LRTI and age-matched controls, and only the second in a low-or middle-income country (LMIC). We used a cohort design with nested case-control analysis to minimize bias. Our data confirm that the pattern of bacterial taxa associated with LRTI (high *Haemophilus*, low *Corynebacterium*, *Dolosigranulum*) is similar to that in high-income countries (HICs) and that the viral pathogens associated with LRTI are similarly conserved. Our analysis of bacterial-viral associations, enabled by the large sample size, suggests that specific interactions are associated with increased risk of LRTI (*Haemophilus* with parainfluenza virus or RSV) or decreased risk of LRTI (*Staphylococcus* with RSV).

### Implications of all the available evidence

Patterns of microbial detection associated with LRTI are similar in a LMIC to that in high-income countries, indicating that these microbes should be targeted to reduce the global burden of LRTI. Preventive interventions should aim to reduce bacterial infection with *Haemophilusinfluenzae* and enhance the growth of the beneficial genera *Corynebacterium* and *Dolosigranulum*. Mechanisms through which Gram-positive bacterial colonization of the nasopharynx may reduce the risk of viral LRTI require further study.
